# Mild Iron Overload as Seen in Individuals Homozygous for the Alpha-1 Antitrypsin Pi*Z Variant Does Not Promote Liver Fibrogenesis in *HFE* Knockout Mice

**DOI:** 10.3390/cells8111415

**Published:** 2019-11-09

**Authors:** Nurdan Guldiken, Karim Hamesch, Shari Malan Schuller, Mahmoud Aly, Cecilia Lindhauer, Carolin V. Schneider, Malin Fromme, Christian Trautwein, Pavel Strnad

**Affiliations:** 1 Medical Clinic III, Gastroenterology, Metabolic Diseases and Intensive Care, University Hospital RWTH Aachen, D-52074 Aachen, Germany; ngueldiken@ukaachen.de (N.G.); khamesch@ukaachen.de (K.H.); shari.schuller@rwth-aachen.de (S.M.S.); maly@ukaachen.de (M.A.); clindhauer@ukaachen.de (C.L.); cheimes@ukaachen.de (C.V.S.); mfromme@ukaachen.de (M.F.); ctrautwein@ukaachen.de (C.T.); 2 Coordinating Center for Alpha-1 Antitrypsin Deficiency-Related Liver Disease of the European Reference Network on Hepatological Diseases (ERN RARE-LIVER) and the European Association for the Study of the Liver (EASL) Registry Group “Alpha-1 Liver”, Germany; 3 Department of Medicine and Infectious Diseases, Faculty of Veterinary Medicine, University of Sadat City, Sadat City 32897, Egypt

**Keywords:** iron metabolism, α1-antitrypsin deficiency, rare liver disease, genetic liver disease, *SERPINA1*, *HFE*, liver fibrosis

## Abstract

The presence of the homozygous ‘Pi*Z’ variant of alpha-1 antitrypsin (AAT) (‘Pi*ZZ’ genotype) predisposes to liver fibrosis development, but the role of iron metabolism in this process remains unknown. Therefore, we assessed iron metabolism and variants in the Homeostatic Iron Regulator gene (*HFE*) as the major cause of hereditary iron overload in a large cohort of Pi*ZZ subjects without liver comorbidities. The human cohort comprised of 409 Pi*ZZ individuals and 254 subjects without evidence of an AAT mutation who were recruited from ten European countries. All underwent a comprehensive work-up and transient elastography to determine liver stiffness measurements (LSM). The corresponding mouse models (Pi*Z overexpressors, *HFE* knockouts, and double transgenic [DTg] mice) were used to evaluate the impact of mild iron overload on Pi*Z-induced liver injury. Compared to Pi*Z non-carriers, Pi*ZZ individuals had elevated serum iron, transferrin saturation, and ferritin levels, but relevant iron overload was rare. All these parameters were higher in individuals with signs of significant liver fibrosis (LSM ≥ 7.1 kPa) compared to those without signs of significant liver fibrosis. *HFE* knockout and DTg mice displayed similar extent of iron overload and of fibrosis. Loss of *HFE* did not alter the extent of AAT accumulation. In Pi*ZZ individuals, presence of *HFE* mutations was not associated with more severe liver fibrosis. Taken together, Pi*ZZ individuals display minor alterations in serum iron parameters. Neither mild iron overload seen in these individuals nor the presence of *HFE* mutations (*C282Y* and *H63D*) constitute a major contributor to liver fibrosis development.

## 1. Introduction

Alpha-1 antitrypsin (AAT) is a major serum protease inhibitor produced predominantly by hepatocytes [1,2]. After its synthesis, AAT is translocated into the endoplasmic reticulum (ER) where it is folded to be secreted into the bloodstream [2]. The most important, disease-causing AAT variant, termed ‘Pi*Z’, interferes with AAT secretion, thereby leading to AAT accumulation in hepatocytes [2,3]. The resulting condition is termed AAT deficiency (AATD) and represents one of the most common genetic disorders potentially leading to death [1,3]. The pathogenic consequences of AAT retention are reproduced in transgenic animals overexpressing the Pi*Z variant (Pi*Z mice; [3]). Similarly to humans, these mice display periodic acid-Schiff-diastase (*PAS*-*D*)-positive AAT globules as well as chronic liver injury that progresses to liver fibrosis [3,4].

In humans, severe AATD is caused mainly by the homozygous Pi*Z mutation (termed ‘Pi*ZZ’ genotype), that is seen in ~1:2000 Caucasians. Pi*ZZ-associated liver disease displays a biphasic pattern and results in clinically relevant liver injury in up to 10% of children and significant liver fibrosis in 20–36% of Pi*ZZ adults [5,6,7,8,9]. Pi*ZZ adults are also strongly susceptible to early-onset emphysema and lung affection constitutes the leading cause of Pi*ZZ-related mortality [2]. Heterozygous Pi*Z carriage (‘Pi*MZ’ genotype) is detected in ~1:40 Caucasians and strongly predisposes to liver cirrhosis in presence of a second hit such as alcoholic and non-alcoholic liver disease [9,10,11].

Although AATD is a monogenic disease, its course is highly heterogeneous and the factors leading to progression of liver disease are largely unknown [8,12]. Several lines of evidence suggested that iron overload may promote liver injury in AATD. In particular, a subset of AATD individuals displayed a moderate to severe hepatic iron overload [13,14]. As a possible explanation, a direct interaction between AAT and hepcidin, the master regulator of iron metabolism, has been described [15]. Additionally, two studies suggested that presence of heterozygous Pi*Z mutation promotes the development of liver fibrosis in individuals with the most common form of hereditary hemochromatosis, that is caused by the homozygous *C282Y* mutation of the Homeostatic Iron Regulator gene (*HFE*) [16,17,18]. Besides affecting iron metabolism, *HFE* mutations, including *C282Y* and the somewhat less pathogenic *H63D* variant, were suggested to lead to ER stress and thereby to increase the proteotoxic injury caused by Pi*Z [19,20].

Similarly, altered iron metabolism was also described in multiple pulmonary diseases including chronic obstructive pulmonary disease (COPD). In the latter one, levels of iron and iron-binding proteins in the lung are increased with normal to reduced systemic iron availability [21,22,23,24]. Moreover, elevated levels of systemic iron are toxic to the lungs and correlate with disease severity and worsening lung function [25,26]. Notably, a genetic variant in iron responsive element binding protein 2 (IREB2), a protein regulating iron levels in the cells, was associated with COPD phenotype in Pi*ZZ individuals [27].

Despite these multiple links, iron metabolism in individuals with severe AATD, i.e., the Pi*ZZ genotype, was never systematically examined. To address this, we analyzed a large international cohort of Pi*ZZ adults for parameters of iron metabolism as well as the presence of *HFE* mutations and directly studied the interaction between mild iron overload and AATD by crossbreeding Pi*Z mice with *HFE* knockouts.

## 2. Material and Methods

### 2.1. Human Cohort

#### 2.1.1. Cohort of Pi*ZZ and Non-Carrier Subjects

In total, 663 adults of self-reported European ancestry were recruited from ten European countries (Austria, Belgium, Denmark, Germany, Italy, Poland, Portugal, Spain, Switzerland, and the Netherlands) in the period from 1 April, 2015 to 31 July, 2019. A major portion of the study population and the recruitment strategy were described previously [7]. The following inclusion criteria were used: (i) age ≥ 18 years, (ii) no known pregnancy, and (iii) the ability to provide a written informed consent. Main exclusion criteria were (i) no presence of genetic material or consent to perform *HFE* mutational analysis, (ii) no availability of serum samples to analyze parameters of iron metabolism, (iii) the presence of a liver comorbidity, (iv) non-valid/not reliable assessment of liver stiffness using transient elastography (TE; FibroScan^®^, Echosens, Paris, France), or (v) non-European descent.

Pi*ZZ subjects (n = 409) were defined as individuals with homozygous carriage of the AAT ‘Pi*Z’ variant (rs28929474, also known as p.E342K or Glu342Lys), i.e., they had the ‘Pi*ZZ’ genotype [7]. Non-carriers (n = 254) were defined as individuals with normal AAT levels (>110 mg/dL) and without evidence of AATD. In all participants, the AAT serum level was determined by nephelometry and genotyping for the most relevant AAT mutations (i.e., the ‘Pi*Z’ variant and the ‘Pi*S’ variant (rs17580)) was carried out [7]. Non-carriers had been recruited from genetically unrelated household members of subjects with an established diagnosis of AATD or as volunteers in liver education campaigns. These campaigns were organized by the University Hospital Aachen (Germany) and were announced via local media to provide a liver examination for the general population [7].

#### 2.1.2. Assessment of Iron Parameters and Exclusion of Concomitant Liver Disease

All comers fulfilling the above mentioned inclusion criteria have been examined and all examinations (surveys, clinical exam, blood sampling, and TE) were done on the same day. Baseline serum samples were used for measurement of the described parameters. Each participant completed standardized questionnaires (e.g., demographic parameters, concomitant diseases, hepatic risk factors, genealogy). As many Pi*ZZ subjects suffer from AATD-related lung disease, lung-related parameters were also assessed (i.e., COPD assessment test (CAT), need of long-term oxygen therapy (LTOT), use of AAT augmentation therapy). In all participants, the presence of a previously existing liver disease was excluded by a personal interview (e.g., no established diagnosis of chronic liver disease, and no history of liver resection or liver transplant) as well as by clinical examination. For each patient, drinking habits were evaluated during a conversation, determining the mean weekly number of alcoholic beverages. Consequently, the amount of alcohol consumed per week was calculated. Individuals with heavy alcohol consumption (≥40 g/day (females) and ≥60 g/day (males)) were excluded (n = 6).

Laboratory workup was performed to exclude the presence of hepatic comorbidities. It consisted of serology to exclude the presence of active hepatitis B or C and a screen for autoimmune hepatitis in individuals with elevated serum transaminases. No participants were tested positive for these parameters.

Serum iron, ferritin, and transferrin levels were measured with the Cobas 8000 analyzer (Roche; Basel, Switzerland) which is approved for commercial testing in the clinical routine. Reference ranges were serum iron, 5.9–34.5 µmol/L [28]; serum ferritin in men, 30–400 ng/mL; serum ferritin in women, 15–150 ng/mL [29]; serum transferrin, 200–360 mg/dL [30]. Transferrin saturation (TSAT) was calculated by an equation using iron and transferrin. TSAT’s reference range was 16–45%.

TE was performed as described to non-invasively determine the extent of liver fibrosis via liver stiffness measurement (LSM) [7]. Notably, TE is recognized as an established method for liver fibrosis assessment [31,32] and was recently validated in two biopsy-proven cohorts of Pi*ZZ adults [6,33]. For LSM, the cut-off of 7.1 kPa was used as a surrogate for significant liver fibrosis (i.e., fibrosis grade≥2) [7]. Participants, in whom different circumstances prevented a reliable LSM assessment (Friedrich-Rust, 2016), were excluded, i.e., (i) individuals with an alanine aminotransferase (ALT) or aspartate aminotransferase (AST) >5× ULN (n = 2) or alkaline phosphatase (ALP) >2× ULN (n = 0); (ii) subjects with less than ten successful TE measurements and an interquartile range ≤30% of the median LSM (n = 26).

#### 2.1.3. *HFE* Mutation Analysis in Pi*ZZ Subjects

Genomic DNA from Pi*ZZ individuals was isolated from whole-blood samples using established DNA isolation kits (Qiagen GmbH, Hilden, Germany and BioBudget Technologies GmbH, Krefeld, Germany) according to the manufacturer’s instruction. *HFE* gene analysis was performed by restriction fragment length polymorphism assay (RFLP) following polymerase chain reaction (PCR) amplification of total genomic DNA of the two regions of the *HFE* gene carrying the mutations *C282Y* and *H63D* [34,35]. Further details are given in the supplementary materials.

#### 2.1.4. Ethical Statement

Ethical approval was granted by the institutional review board of RWTH Aachen University (EK 173/15) as well as by the institutional ethics committees at each study center in the participating countries. All participants gave a written informed consent form and the study was conducted following the ethical guidelines of the Helsinki Declaration (Hong Kong Amendment) as well as Good Clinical Practice (European guidelines). The study was registered with ClinicalTrials.gov (NCT029292940).

### 2.2. Mouse Experiments

Previously established transgenic mice overexpressing the mutant human Pi*Z protein (PiZ) [36] or lacking the Homeostatic Iron Regulator gene (*HFE*) [37,38] were crossbred and genotyped as described ([38], Jackson Laboratory 2016). Age-matched WT littermates were used as controls. Mice were sacrificed at the age of 3 and 18 months, and blood was collected by intrahepatic aspiration to measure liver enzymes (ALT, AST, ALP) and iron parameters. Livers were removed and cut into pieces for (i) fixation in 4% formaldehyde for histological staining and (ii) snap freezing for hepatic iron concentration, hydroxyproline, and immunoblotting analysis. Details on staining and biochemical methods are given in the supplementary materials. All animals received humane care and their use was approved by the Institutional Animal Care Committee.

### 2.3. Statistical Analysis

All categorical variables were presented as absolute (n) and relative (%) frequencies and the corresponding contingency tables were analyzed with Chi square tests. Continuous variables were described as mean ± standard deviation (SD). For human analyses, continuous variables were analyzed by unpaired, two-tailed t-tests as well as by a multivariable linear model to account for relevant confounders. Adjustments were made for age, sex, body mass index (BMI), presence of diabetes mellitus, and mean daily alcohol consumption as described previously [7]. For murine analyses, continuous variables were analyzed by a parametric 1-way ANOVA with Newman–Keuls post hoc test. Nominal *p* values were given for all statistical tests and differences were considered to be statistically significant when *p* < 0.05. The data were analyzed using SPSS Statistics version 23 (IBM; Armonk, NY, USA) and Prism version 5 (GraphPad, LaJolla, CA, USA).

## 3. Results

### 3.1. Pi*ZZ Individuals Displayed Mildly Elevated Iron Parameters

To determine the impact of severe AAT deficiency on iron metabolism, we analysed a cohort of 409 Pi*ZZ subjects and 254 non-carriers. Pi*ZZ individuals were significantly older and as expected, presented with impaired lung function as well as higher surrogate parameters of liver fibrosis (liver stiffness measurements, LSM) and steatosis (controlled attenuation parameter, CAP) (Table 1).

**Figure 1:**
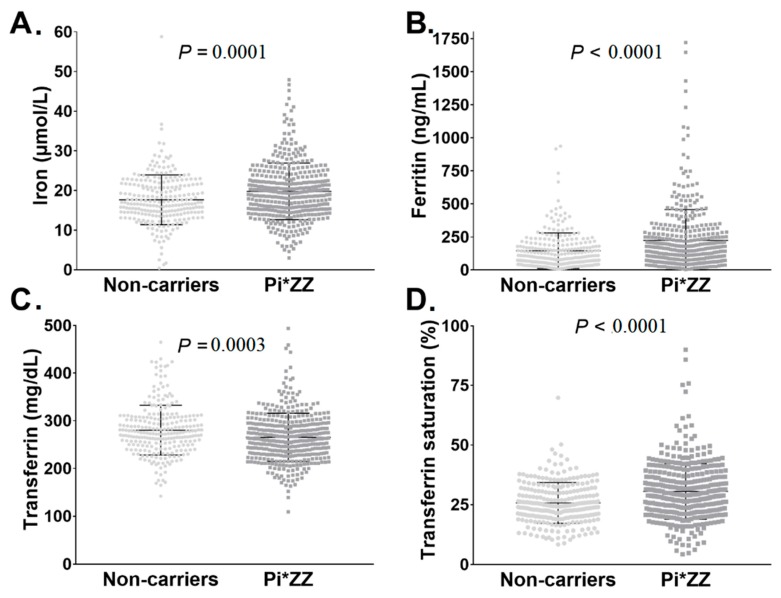
Parameters of iron metabolism in individuals homozygous for the alpha1-antitrypsin Pi*Z variant (Pi*ZZ) and Pi*Z non-carriers. In total, 254 non-carriers and 409 individuals with a homozygous Pi*Z mutation (Pi*ZZ) were analyzed. Scatter plots depict serum iron (adjusted *p* value = 0.0002) (**A**), serum ferritin (adjusted *p* value = 0.000003) (**B**), serum transferrin (adjusted *p* value = 0.0037) (**C**), and serum transferrin saturation (adjusted *p* value = 1.0 × 10^−7^) (**D**). Multivariable adjustments were performed for the covariates age, sex, BMI, presence of diabetes mellitus, and mean alcohol consumption.

Although parameters of iron metabolism were mostly within the reference range in both groups (Figure 1), serum iron, ferritin, and TSAT were significantly higher in Pi*ZZ subjects vs. non-carriers, whereas serum transferrin was lower (Figure 1). Only a small fraction of Pi*ZZ individuals (8/409, 2%) had serum ferritin values >1000 ng/mL. Notably, these patients had a median LSM of 11.0 kPa suggestive of advanced liver fibrosis.

While we excluded individuals with harmful alcohol consumption (women: ≥40 g/d, men: ≥60 g/d) *a priori*, we wondered whether the observed differences in iron parameters might be due to individuals with “intermediate” alcohol consumption (defined as 21–40g/d in women and 31–60g/d in men). 34/663 individuals (5%) in the overall cohort reported this alcohol behavior (17 in each subcohort). However, the exclusion of these individuals and the consequent use of more stringent cut-offs yielded identical differences in iron-related parameters between Pi*ZZ individuals and non-carriers (Figure S1).

**Figure 2:**
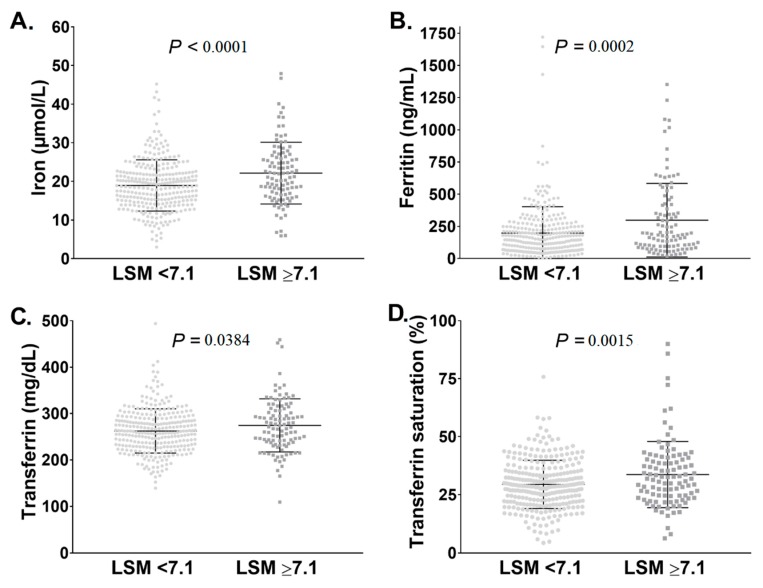
Parameters of iron metabolism in subjects homozygous for the Pi*Z variant (Pi*ZZ) with and without liver stiffness measurements (LSM) suggestive of significant liver fibrosis. Pi*ZZ individuals (n = 409) were subjected to non-invasive assessment by transient elastography (FibroScan^®^). A previously described cut-off of 7.1 kPa for LSM [7], suggestive of the presence of significant liver fibrosis, was used. Serum iron (adjusted *p* value = 0.0002) (**A**), serum ferritin (adjusted *p* value = 0.0106) (**B**), serum transferrin (adjusted *p* value = 0.0211) (**C**), and serum transferrin saturation (adjusted *p* value = 0.0080) (**D**) values were compared between Pi*ZZ subjects with LSM below and above 7.1 kPa. Multivariable adjustments were performed for the covariates age, sex, BMI, presence of diabetes mellitus, and mean alcohol consumption.

Since altered iron metabolism is commonly seen in advanced liver fibrosis [39], we assessed serum iron parameters in Pi*ZZ individuals with versus without significant liver fibrosis determined as LSM ≥ 7.1 and <7.1kPa, respectively. Notably, serum iron, ferritin, transferrin, and TSAT were all significantly higher in Pi*ZZ individuals with LSM ≥ 7.1 compared to LSM < 7.1kPa. (Figure 2).

As altered parameters of iron metabolism can be seen in hepatic steatosis, we compared serum iron parameters in Pi*ZZ individuals with vs. without non-invasive signs of severe steatosis (cut-off for CAP of 280 dB/m), respectively [40]). Serum ferritin was higher in Pi*ZZ subjects with CAP ≥ 280 dB/m compared to those with CAP < 280 dB/m in the univariable analysis, but the difference got lost after adjustment for age, sex, BMI, diabetes mellitus, and alcohol consumption (Figure S2A). The other parameters of iron metabolism (i.e., iron, transferrin, and transferrin saturation) were not different in Pi*ZZ individuals with or without CAP ≥ 280 dB/m, both in the univariable and multivariable analysis (Figure S2A–D). Serum ferritin, as the only altered parameter of iron metabolism in steatotic Pi*ZZ individuals, correlated weakly with CAP (rho = 0.20, *P* = 0.0001; Figure S2E). Likewise, CAP and LSM correlated weakly with each other (in Pi*ZZ carriers: rho = 0.22, *p* < 0.0001; Figure S2F).

To assess whether the presence of lung disease has any impact on iron metabolism, iron parameters were compared in Pi*ZZ individuals with high vs. low CAT scores and with vs. without long-term oxygen therapy (LTOT) (Figures S3 and S4). Apart from serum ferritin, which was significantly higher in Pi*ZZ individuals with higher CAT scores, no alterations in iron parameters were noted (Figures S3 and S4). In summary, we demonstrated that Pi*ZZ subjects display mildly increased parameters of iron metabolism and that these associate with impaired liver rather than lung function.

### 3.2. Mild Iron Overload in Double Transgenicmice Did Not Alter the Course of Pi*Z-Associated Liver Disease

**Figure 3:**
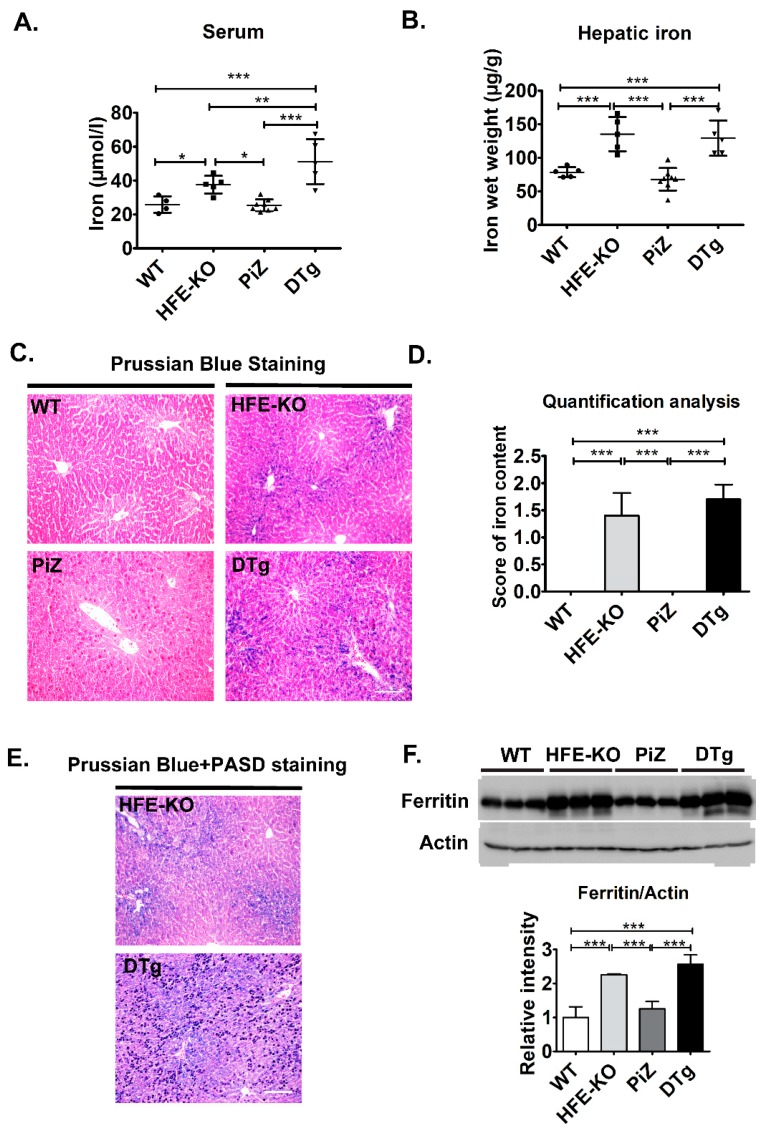
Overexpression of the Pi*Z variant of the human alpha1-antitrypsin (AAT) did not lead to iron accumulation in three months old mice. Serum (**A**) and hepatic (**B**) iron levels were measured and Prussian Blue staining (**C**) with morphometric quantification (**D**) was carried out in non-transgenic (WT) and Homeostatic Iron Regulator knockout (*HFE*-KO) mice, mice overexpressing the Pi*Z variant of human AAT (PiZ) as well as double transgenic animals (DTg). Combined periodic acid-Schiff-diastase (PASD) and Perls Prussian Blue stainings (**E**) visualize the location of iron and AAT deposits. Immunoblotting and subsequent morphometric quantification assessed hepatic ferritin levels compared to actin, that was used as a loading control (**F**). Results are shown as mean ± SD (n ≥ 4 per group). Scale bar: 100 µm. * *p* < 0.05, ** *p* < 0.01, *** *p* < 0.001.

To test whether the minor increase in iron parameters seen in Pi*ZZ individuals may affect the development of Pi*ZZ-related liver disease, we crossbred Pi*Z-overexpressing mice with *HFE* knockouts (*HFE*-KO). At three months of age, both PiZ and DTg mice had slightly elevated liver-to-body weight ratios, whereas no differences in body weight or serum enzyme levels were noted (Table 2). In line with the latter, the animals did not display any obvious histological signs of liver damage (not shown). As expected, moderately increased serum iron levels were seen in *HFE*-KO and DTg animals with the latter group displaying the highest values (Figure 3A). Hepatic iron levels were determined biochemically as well as morphometrically after Prussian blue staining and both methods revealed a similar extent of iron overload in *HFE*-KO animals and DTgs when compared to WT and Pi*Z-overexpressing mice (Figure 3B–D).

**Figure 4:**
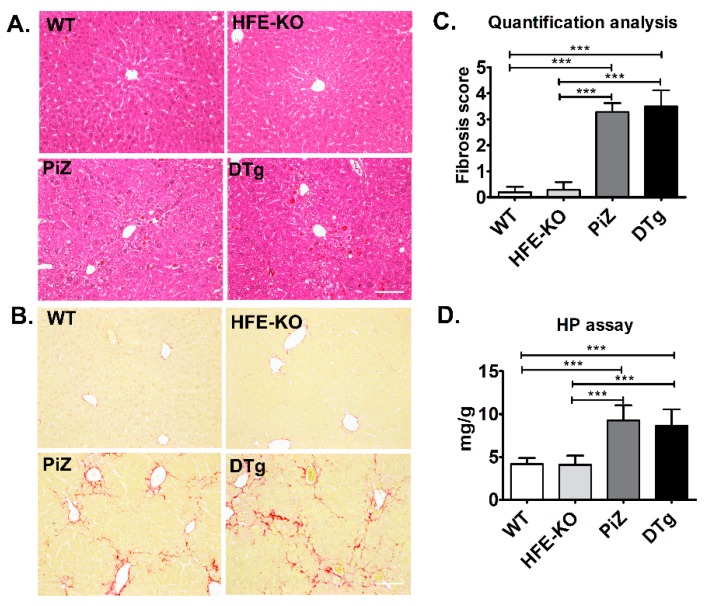
Mild iron overload did not accelerate the development of liver fibrosis in transgenic mice overexpressing the Pi*Z variant of human alpha1-antitrypsin (AAT). Hematoxylin & Eosin (H&E) (**A**) and Sirius red stainings (**B**) with morphometric quantification (**C**) were performed on livers from 18 month-old non-transgenic (WT) and Homeostatic Iron Regulator knockout (*HFE*-KO) mice, mice overexpressing the Pi*Z variant of the human AAT (PiZ) as well as double transgenic animals (DTg). The extent of liver fibrosis was also assessed biochemically via measurement of hydroxyproline (HP) content (**D**). Results are presented as mean ± SD (n ≥ 5). * *p* < 0.05, ** *p* < 0.01, *** *p* < 0.001.

To visualize the relationship between iron deposits and Pi*Z inclusions, we performed a double staining with PASD and Prussian blue (Figure 3E). In line with previous reports [37], iron deposits were found predominantly in the periportal area, while Pi*Z inclusions were scattered throughout the whole liver lobe. Notably, neither Pi*Z nor iron deposition pattern were visibly altered in DTg animals (Figure 3E). This impression was confirmed by a morphometric quantification of PASD staining as well as by immunoblotting of total tissue lysates, that both revealed no differences in Pi*Z accumulation between PiZ and DTg mice (Figure S5). Finally, *HFE* knockouts and DTg animals displayed increased, but comparable hepatic ferritin levels, thereby further demonstrating that presence of Pi*Z does not affect the hepatic iron accumulation (Figure 3F). To test whether iron overload affects the development of Pi*Z-associated liver fibrosis, we turned to 18 month-old animals. Both PiZ and DTg mice displayed significantly lower, but comparable body weights as well as similarly increased serum ALP levels (Table 3). Even at this age, WTs and *HFE*-KOs had largely intact liver architecture, whereas the presence of large Pi*Z inclusions, somewhat distorted lobe structure as well as liver fibrosis was noted in PiZ and DTg mice (Figure 4A,B). The extent of liver fibrosis was similar in PiZ and DTg animals, as demonstrated both via morphometric analysis and quantification of hepatic hydroxyproline content (Figure 4C,D). Compared to younger animals, 18 months old mice displayed less numerous, larger Pi*Z inclusions (Figure S6A). Neither morphometric analysis nor immunoblotting with an AAT antibody revealed any differences in AAT retention between PiZ and DTg mice (Figure S6). Taken together, a mild iron overload in DTg mice did have an obvious impact neither on the extent of AAT accumulation nor on the development of liver fibrosis.

### 3.3. Presence of HFE Mutations Did Not Associate with Increased Liver Injury in Pi*ZZ Individuals

While our above analyses focused on loss of *HFE*, we also tested, whether presence of the *HFE* mutations *H63D* and *C282Y* alters the liver phenotype of Pi*ZZ subjects. Both variants were found at frequencies comparable to the ones reported for Caucasian population and were not significantly different from the expectations of Hardy–Weinberg equilibrium [18]. None of the analyzed Pi*ZZ subjects was homozygous for the *C282Y* variant. Apart from the compound heterozygous *HFE* genotype that associated with somewhat increased TSAT values, no alterations in iron metabolism were noted. However, only 4/380 (1.1%) were compound heterozygous. Moreover, subgroups with different *HFE* haplotypes displayed comparable LSM values (Table 4). In summary, although we cannot exclude a potentially detrimental effect of a homozygous *C282Y* variant, common *HFE* mutations do not seem to exacerbate the liver phenotype of Pi*ZZ individuals.

## 4. Discussion

In our study, we systematically assessed iron parameters in a large cohort of Pi*ZZ individuals as well as Pi*Z-overexpressing mice crossbred with *HFE*-KOs. While the observed changes in iron parameters were small, we detected a consistent increase in serum iron levels in Pi*ZZ individuals vs. non-carriers as well as in DTg mice vs. PiZ animals. As hepcidin is the primary negative regulator of serum iron, diminished hepcidin production might be responsible for this observation. In fact, AAT induces hepcidin expression and the decreased serum AAT levels seen in Pi*ZZ individuals may therefore result in lower hepcidin values [14]. In addition, decreased hepcidin production was seen in multiple liver disorders [39] and this stress-induced suppression may translate to Pi*ZZ livers. This scenario was further supported by the fact that LSM ≥ 7.1kPa, suggestive of significant liver fibrosis, was associated with a further increase in serum iron levels in Pi*ZZ subjects. A similar, stress-driven mechanism may also apply to serum transferrin, that was decreased in sera from Pi*ZZ adults vs. non-carriers. Notably, transferrin is a negative acute phase reactant that is suppressed in various stress conditions [3]. Moreover, transferrin transcription is driven via *HNF4* signaling and this pathway is down-regulated in Pi*Z-overexpressing mice [32,41,42].

Pi*ZZ adults also had somewhat higher serum ferritin values than non-carriers. This finding might be both because of a higher iron load and/or increased liver injury since ferritin is produced predominantly in the liver and becomes released during liver damage [43]. The latter mechanism might be particularly relevant in Pi*ZZ individuals with LSM ≥ 7.1kPa, that display the highest ferritin values. While clinically relevant ferritin increase (>1000 ng/mL), that is associated with an advanced risk of liver fibrosis in hereditary hemochromatosis [18], was uncommon (2% of Pi*ZZ subjects), it was associated with elevated LSM values. Therefore, a combination of both variants might be detrimental in a small subset of affected people and these individuals need a close clinical follow-up [14]. Notably, our findings cannot delineate whether increased iron is a cause or consequence of liver fibrosis in these Pi*ZZ individuals.

While the presence of significant liver fibrosis was associated with clear alterations in iron parameters, the occurrence of advanced lung disease did not significantly affect the parameters of iron metabolism. This finding is somewhat surprising since hypoxia is a well-established suppressor of hepcidin production [44] and since genetic variants in *IREB2* associate with the COPD phenotype in Pi*ZZ individuals. In that respect, two important limitations of our study must be stressed: (i) our cohort provides only a limited lung phenotyping and (ii) our work did not assess genes regulating iron levels and distribution within the cells. Therefore, further studies are needed to uncover more subtle phenotypic consequences in the lung as well as to fully explore the wider iron pathways.

Given the above described findings, we decided to use *HFE*-KO mice as a model of mild iron overload. Notably, these animals are well-suited for this goal, since they, similarly to Pi*ZZ individuals, display a mild increase in serum iron and ferritin values as well as an increased transferrin saturation [37]. In the resulting DTg mice, we did not observe any signs of increased injury/fibrosis nor any obvious alterations in AAT metabolism. These data suggest that the minor alterations in iron metabolism occurring in the majority of Pi*ZZ individuals do not contribute to liver disease progression. However, we cannot exclude potentially detrimental consequences of severe iron overload that arises in a small subset of AATD individuals. This may also apply to individuals with a homozygous *HFE C282Y* or compound heterozygous *C282Y/H63D* genotype, that were not or only rarely found in our study, respectively.

Apart from iron overload, the presence of an *HFE* mutation leads to ER stress [19,45] and this type of injury is particularly relevant in Pi*ZZ individuals, that display an accumulation of polymerized AAT in their ERs [3]. To test the interaction between both hits, we genotyped our Pi*ZZ cohort for the *H63D* and *C282Y* variants of the *HFE* gene, but the detected haplotypes did not show obvious differences in their LSM values. However, the haplotypes with higher risk of liver fibrosis development did either not occur (*C282Y* homozygosity) or were rare (four individuals with *C282Y*/*H63D* compound heterozygosity) and further studies are needed to clarify their impact. Nevertheless, the presence of heterozygous *H63D* and *C282Y* variants, either alone or in combination does not seem to relevantly exacerbate the Pi*ZZ-associated liver injury. However, since we analyzed an adult cohort, we cannot exclude an effect of such variants on pediatric liver disease, that has been suggested in a previously published study [20]. Moreover, we cannot evaluate the impact of *C282Y* homozygosity, that occurs in <1% of the Caucasian population. Collectively, these data indicate that a mild iron overload can be tolerated in Pi*ZZ individuals, while markedly increased values of iron metabolism should trigger a corresponding clinical workup.

## Figures and Tables

**Table 1:** Characteristics of individuals homozygous for the alpha-1 antitrypsin Pi*Z variant (Pi*ZZ) and subjects without Pi*Z carriage. Quantitative measures are expressed as mean ± standard deviation or as relative frequency (%).

Category	Non-Carriers (n = 254)	Pi*ZZ Carriers (n = 409)	*p* Value
Age (years)	51.8 ± 15.4	56.1 ± 12.2	<0.0001
Women (%)	50.8	46.0	0.23
BMI (kg/m^2^)	25.5 ± 4.6	25.0 ± 4.3	0.30
BMI ≥ 30 kg/m^2^ (%)	17.8	15.7	0.48
Diabetes (%)	5.1	3.4	0.28
Mean alcohol consumption (g/d)	7.5 ± 10.3	6.0 ± 9.5	0.15
COPD assessment test (points)	7.6 ± 6.4	17.3 ± 8.8	<0.0001
Long-term oxygen therapy (%)	0.4	21.1	<0.0001
AAT augmentation therapy (%)	0	60.4	<0.0001
Liver stiffness (kPa) *	4.7 ± 2.8	7.1 ± 7.3	<0.0001
Controlled attenuation parameter (dB/m)	245 ± 59	268 ± 60	<0.0001

* After multivariable adjustments for age, sex, BMI, diabetes, and mean alcohol consumption the adjusted *p* value was 0.000013. Abbreviations: AAT, alpha-1 antitrypsin; BMI, body mass index; COPD, chronic obstructive pulmonary disease.

**Table 2:** Liver values, serum parameters, and body weights in three months old mice.

Experimental Group	WT	HFE-KO	PiZ	DTg
Body weight (g)	22.9 ± 1.04	23.4 ± 1.6	22.3 ± 1.5	22.8 ± 1.7
Liver/Body weight (g)	0.04 ± 0.002 ^a,b^	0.04 ± 0.004 ^c,d^	0.05 ± 0.004 ^a,c^	0.05 ± 0.005 ^b,d^
ALT (U/L)	21 ± 6.3	18.6 ± 5.4	25.5 ± 6	24.6 ± 3.9
AST (U/L)	42.6 ± 11.1	48.6 ± 6.5	57.7 ± 13.5	56.4 ± 17.3
ALP (U/L)	99.6 ± 40.7	77.4 ± 9.1	84.4 ± 11.3	99.6 ± 46.3

WT, wild type; *HFE*-KO, mice deficient in Homeostatic Iron Regulator gene; PiZ, mice overexpressing the human Pi*Z variant; DTg, double transgenic mice. Values are expressed as mean ± SD (n ≥ 5 per group). *p* values (ANOVA, Newman–Keuls); ^a,b^ *p* = **, ^c,d^ *p* = *. * *p* < 0.05, ** *p* < 0.01, *** *p* < 0.001.

**Table 3:** Liver values, serum parameters and body weights in 18 months old mice.

Experimental Group	WT	*HFE*-KO	PiZ	DTg
Body weight (g)	33.2 ± 2.9 ^a,b^	32.8 ± 3.6 ^c,d^	27.8 ± 1.5 ^a,c^	26.4 ± 2.4 ^b,d^
Liver/Body weight (g)	0.04 ± 0.004	0.05 ± 0.01	0.05 ± 0.004	0.05 ± 0.005
ALT (U/L)	49 ± 18.8	75.6 ± 48.6	56.6 ± 18.6	51.2 ± 20.6
AST (U/L)	79.8 ± 32.3	106 ± 48.6	122.7 ± 29	118.1 ± 26.5
(U/L)	105.8 ± 47.2 ^e,f^	94.2 ± 32 ^g,h^	204 ± 68.8 ^e,g^	189.4 ± 76.7 ^f,h^

WT, wild type; *HFE*-KO, mice deficient in Homeostatic Iron Regulator gene; PiZ, mice overexpressing the human Pi*Z variant; DTg, double transgenic mice; Values are expressed as mean ± SD (n ≥ 5 per group). *p* values (ANOVA, Newman–Keuls); ^a,b,c,d^ *p* < 0.01, ^e,f,g,h^ *p* < 0.05

**Table 4:** Characteristics of Pi*ZZ subjects subdivided based on presence of *C282Y* and *H63D* variants in the *HFE* gene. None of the patients was homozygous for the *C282Y* variant, hence this group was not shown. Quantitative measures are expressed as mean ± standard deviation or as relative frequency (%).

Category	C282Y & H63D Non-Carriers (n = 246)	C282Y Hetero-zygous (n = 24)	H63D Hetero-zygous (n = 99)	H63D Homo-zygous (n = 7)	C282Y/H63D Compound Heterozygous (n = 4)
Age (years)	56.5 ± 12.1	53.1 ± 13.8	56.0 ± 11.7	55.6 ± 15.9	65.7 ± 11.1
Women (%)	44.3	50.0	50.5	42.9	46.3
BMI (kg/m^2^)	24.9 ± 3.8	25.8 ± 5.0	25.2 ± 5.4	23.8 ± 2.3	23.5 ± 2.2
BMI ≥ 30 kg/m^2^ (%)	14.3	20.8	20.2	0	0
Diabetes (%)	2.8	0	6.1	0	25.0
Mean alcohol consumption (g/d)	6.6 ± 9.6	2.6 ± 3.5	5.3 ± 10.3	2.9 ± 4.4	3.0 ± 5.9
COPD assessment test (points)	17.3 ± 8.9	15.8 ± 8.5	18.7 ± 8.3	14.4 ± 13.2	13.2 ± 8.2
Long-term oxygen therapy (%)	20.8	20.8	24.5	14.3	25.0
AAT augmentation therapy (%)	61.1	50.0	64.6	57.1	50.0
Ferritin (ng/mL)	205.8 ± 203.2	160.7 ± 154.6	246.9 ± 228.3	167.6 ± 93.9	238.0 ± 160.8
Transferrin (mg/dL)	268.8 ± 55.3	259.6 ± 37.9	261.4 ± 3.6	247.7 ± 57.2	233.8 ± 43.1
TSAT (%) *	29.2 ± 10.4	33.9 ± 17.1	30.3 ± 9.3	38.3 ± 8.9	57.8 ± 20.1
Liver stiffness (kPa)	7.1 ± 6.7	6.0 ± 2.8	5.9 ± 3.1	5.1 ± 1.3	6.4 ± 3.7
LSM ≥ 7.1 kPa (%)	25.6	22.7	20.4	14.3	25.0
LSM ≥ 10 kPa (%)	13.0	9.1	5.1	0	25.0

* Significant *p* values for TSAT: C282Y/H63D non-carriers vs. compound heterozygotes: unadjusted *p* < 0.0001, adjusted *p* < 0.0001. Multivariable adjustments were performed for the covariates age, sex, BMI, presence of diabetes mellitus, and mean alcohol consumption. Abbreviations: AAT, alpha-1 antitrypsin; BMI, body mass index; COPD, chronic obstructive pulmonary disease; LSM, liver stiffness measurement.

## Supplementary Materials

The following are available online at https://www.mdpi.com/2073-4409/8/11/1415/s1, Table S1: Mouse and human genotyping primers, Figure S1: Parameters of iron metabolism in individuals homozygous for the alpha1-antitrypsin Pi*Z variant (Pi*ZZ) and Pi*Z non-carriers, both with low alcohol consumption (i.e., ≤30 for men and ≤20 for women). Figure S2: Iron parameters in Pi*ZZ carriers with and without non-invasive signs of severe liver steatosis. Figure S3: Parameters of iron metabolism in Pi*ZZ individuals with and without long-term oxygen therapy (LTOT). Figure S4: Parameters of iron metabolism in Pi*ZZ subjects with low vs. high COPD assessment test (CAT) scores. Figure S5: Mild iron overload does not alter the amount of alpha1-antitrypsin (AAT) accumulation in 3 months-old mice overexpressing the Pi*Z variant of AAT. Figure S6: Mild iron overload does not alter the amount of alpha1-antitrypsin (AAT) accumulation in 18 months-old mice overexpressing the Pi*Z variant of AAT.

## Abbreviations

AAT	alpha-1 antitrypsin
AATD	Alpha-1 antitrypsin deficiency
ALT	alanine aminotransferase
AST	aspartate aminotransferase
CAP	Controlled attenuation parameter (via transient elastography (FibroScan^®^)
CAT	COPD assessment test
COPD	chronic obstructive pulmonary disease
*HFE*	Hereditary Hemochromatosis Protein
LSM	liver stiffness measurement (via transient elastography (FibroScan^®^)
LTOT	long-term oxygen therapy
*SERPINA1*	serpin family A member 1 (AAT gene)
TF	transferrin
TSAT	transferrin saturation
