# Supplementary material for: Mild Iron Overload as Seen in Individuals Homozygous for the Alpha-1 Antitrypsin Pi*Z Variant Does Not Promote Liver Fibrogenesis in HFE Knockout Mice
Source: Cells. 2019 Nov 9;8(11):1415. doi: 10.3390/cells8111415 (PMC6912453; doi:10.3390/cells8111415)
Supplement: Supplementary file 1 [file cells-08-01415-s001.pdf]

Online-only supplement of Guldiken N*, Hamesch K* et al. – Mild iron overload as seen in individuals homozygous for the alpha-1 antitrypsin Pi*Z variant does not promote liver fibrogenesis in *HFE* knockout mice

Supplementary Methods:

***HFE* mutation detection in human samples**

To detect the *H63D and C282Y* mutations, a 393 base pair (bp) and 208 bp products were amplified with previously described primers (Table S1)[27, 28]. PCR amplification was carried out with a commercial master mix (BioBudget Technologies GmbH, Germany) and 0,1–1 µm of each primer. Subsequently, the amplicons were digested with *RsaI* restriction endonuclease (R0167, New England Biolabs,Germany; detection of *C282Y* variant) [38] or *BclI* restriction endonuclease (R0160, New England Bioloabs; detection of *H63D* variant) [28, 39] as recommended by the manufacturer. *Rsa*I cleavage of wild-type allele produces 247-bp and 146-bp fragments, whereas the presence of the *C282Y* mutation results in an additional cleavage of the 146-bp fragment into 117 bp and 29 bp products. *BclI* cleaves the 208-bp PCR product into two fragments of 138 and 70 bp length in the wild-type, but does not digest in the mutant allele. The fragments were resolved by electrophoresis in a 3% agarose gel.

Table S1. Mouse and human genotyping primers.

**Gene**	**5′- Sequence -3′**	**Accession number**
Transgenic mice		
Serpina1a (AAT)	F:GCAGCCTGACTTCTTTGTGC<br>R:ATCCTAGGGGGCTTGGTGAT	**NC_000014.9**
Homeostatic Iron Regulator (*HFE*)	WT F: GGGAAATGTTAGTGGCCTGA<br>WT R: AGTGGCGAGTCACTTTCACC<br>KO F:AGTTGGGAGTGGTGTCCGA<br>KO R:CTAGCTTCGGCCGTGACG	**NC_000079.6**
HFE mutations (human)		
HFE C282Y	F: TGGCAAGGGTAAACAGATCC<br>R: TACCTCCTCAGGCACTCCTC	**NC_000006.12**
HFE H63D	F: ACATGGTTAAGGCCTGTTGC<br>R: GCCACATCTGGCTTGAAATT	

F, forward; KO, knockout; R, reverse; WT, wild type

Tissue stainings

For histological staining, the harvested liver tissues were kept in 4% buffered formalin at room temperature overnight. Afterwards, they were removed, dehydrated, embedded in paraffin, cut onto 2 µm thick sections and stained with Hematoxylin and Eosin, Perls Prussian Blue (iron staining), Sirius Red staining, and Periodic acid – Schiff diastase staining as specified below.

Hematoxylin and eosin staining (H&E) was used to analyze the extent of liver injury. Sections were first deparaffinized with xyline and hydrated with a serial dilution of ethanol following washing steps with water. The basophilic nuclei were stained with a hematoxylin (blue color) and the acidophilic cytoplasm was stained with eosin (pink) [40].

Sirius Red staining was used to visualize the collagen fibers and thereby to quantify the extent of liver fibrosis. After deparaffinization in xylol, the sections were hydrated with serial dilutions of isopropanol. After washing shortly with deionized water, they were stained in Sirius red solution for 1 hour at room temperature [40]. Fibrosis scores were determined by modified Ishak scoring system: score 0-no obvious pathology; score 1-mild; periportal; score 2-moderate; portal fibrosis; score 3-distinct; septal fibrosis; score 4-complete bridging fibrosis. Iron distribution was assessed via modified Deugnier score as described previously [41]: 0-granules absent; 1-granules barely discernible; 1.5-granules discernible; 2-masses visible.
Periodic acid – Schiff diastase staining (PASD) was used to visualize and analyze AAT inclusions. Firstly, the deparaffinized sections were hydrated with serial dilutions of isopropanol and washed shortly with deionized water. To degrade polysaccharides, the tissues were treated with saliva which contains abundant amount of α-amylase (also known as diastase) for 30 minutes at RT. After treatment with periodic acid (P0430; Sigma, Steinheim, Germany) solution, subsequent incubation with Schiff reagent (Sigma, Germany) for 15 minutes at RT resulted in positive staining of magenta colored–diastase resistant AAT globules that were analyzed microscopically. To quantify the extent of AAT inclusions, stained liver sections were scored by two independent observers as: 0, no inclusions; 1-mild; 1.5-moderate; 2-distinct in periportal areas.

Protein analysis

Total liver lysates were prepared by homogenization in 3 % sodium dodecyl sulphate (SDS, Roth, Germany) containing sample buffer followed by centrifugation to remove non-soluble debris. The isolated protein extracts were diluted in 4x reducing Laemmli buffer and the proteins were separated by 10% SDS–polyacrylamide gel electrophoresis. Electrophoresed samples were either visualized by Coomassie staining or were transferred to PVDF membranes (GE Healthcare, Germany) for immunoblotting. Membranes were blocked and incubated with the appropriate primary and secondary antibodies, the resulting HRP signal was visualized by enhanced chemiluminescence (GE Healthcare, UK). Following primary antibodies were used: human AAT (D11, kindly provided by Prof. Dr. Janciauskiene [42]), mouse ferritin (FTH1; 3998, Cell signaling, Germany) and mouse GAPDH (NB300-221; NovusBio, UK)

Biochemical assays

Quantification of hepatic collagen content was performed with a hydroxyproline assay as described [43]. Briefly, liver tissue was homogenized in distilled water and hydrolyzed in 6N HCL at 110 °C for 18 hours. Lysates were filtered (55 mm, Macherey-Nagel, Germany) to remove debris and evaporated by speed vacuum centrifugation. The pellets or standards (trans-4-hydroxy-L-proline; H54409, Sigma, Steinheim, Germany) were dissolved in distilled water, then mixed with 0,6% of chloramines-T (31224, Sigma, Steinheim, Germany) for 10 minutes at RT in order to oxidize free hydroxyproline to pyrrole. Ehrlich solution (X867.1, Roth, Karlsruhe, Germany) was added to form the desired chromophore and measured at wavelength of 570 nm using a plate reader (BioTek Cytation 3, USA). Hydroxyproline content was normalized to wet liver weight and was presented as mg/g (hydroxyproline amount / wet liver weight).
To quantify the extent of iron accumulation, hepatic non-heme iron content was measured as described [43]. Briefly, samples were incinerated at 110 °C for 24 hours and hydrolysed in 100mM citric acid (C0759, Sigma, Steinheim, Germany) at 60 °C for 4 hours. After reduction with L-ascorbic acid (A5960, Sigma, Steinheim, Germany), bathophenanthroline disulfonic acid disodium salt (BPT, 146617, Sigma, Steinheim, Germany) was added that forms a red color complex upon reaction with iron. Absorbance was measured with a spectrophotometer at 535 nm via a plate reader (BioTek Cytation 3, USA). The normalization was done according to wet liver weight and was presented as μg/g (iron amount / wet liver weight).

Figures

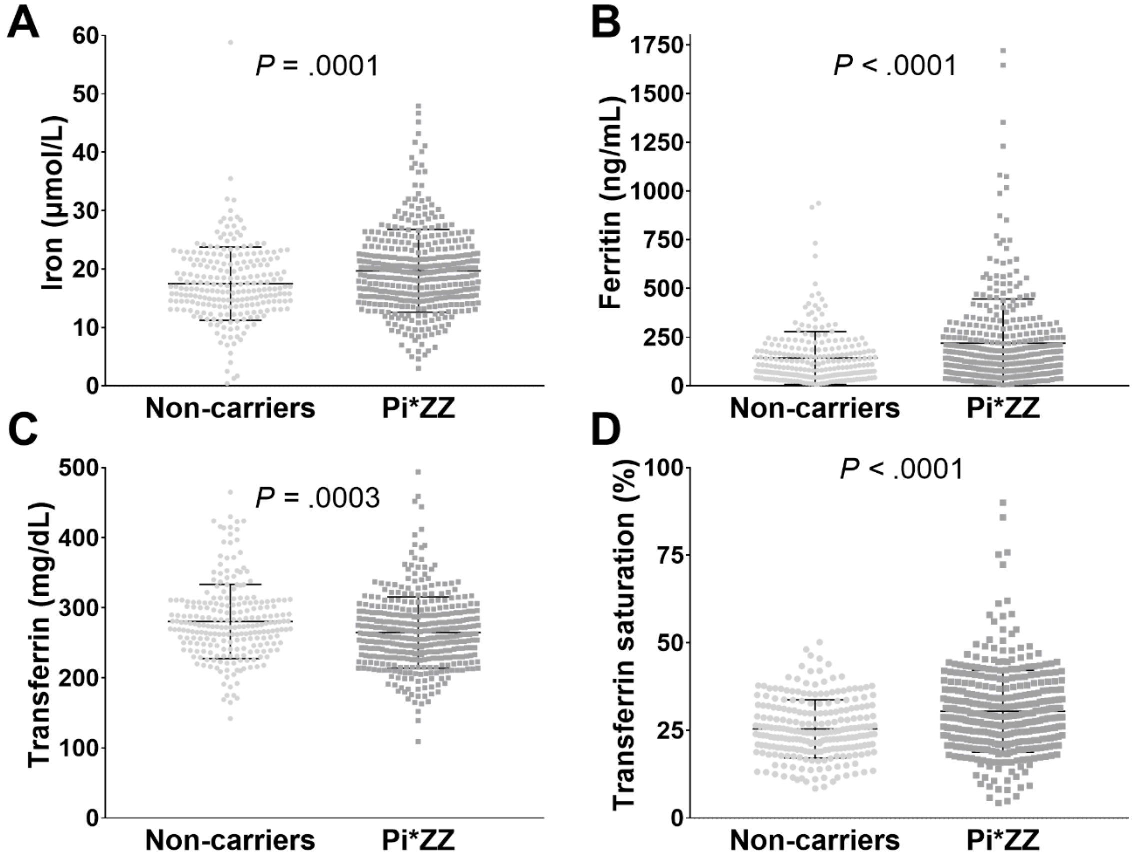

Figure S1. Parameters of iron metabolism in individuals homozygous for the alpha1-antitrypsin Pi*Z variant (Pi*ZZ) and Pi*Z non-carriers, both with low alcohol consumption (i.e. ≤30 for men and ≤20 for women). 237 non-carriers and 392 Pi*ZZ individuals were analyzed. Scatter plots depict serum iron (adjusted *P* value=0.0002) **(A)**, serum ferritin (adjusted *P* value=0.00002) **(B)**, serum transferrin (adjusted *P* value=0.0030) **(C)**, and serum transferrin saturation (adjusted *P* value=1.2×10^{-7}) **(D)**. Multivariable adjustments were performed for the covariates age, sex, BMI, presence of diabetes mellitus, and mean alcohol consumption.

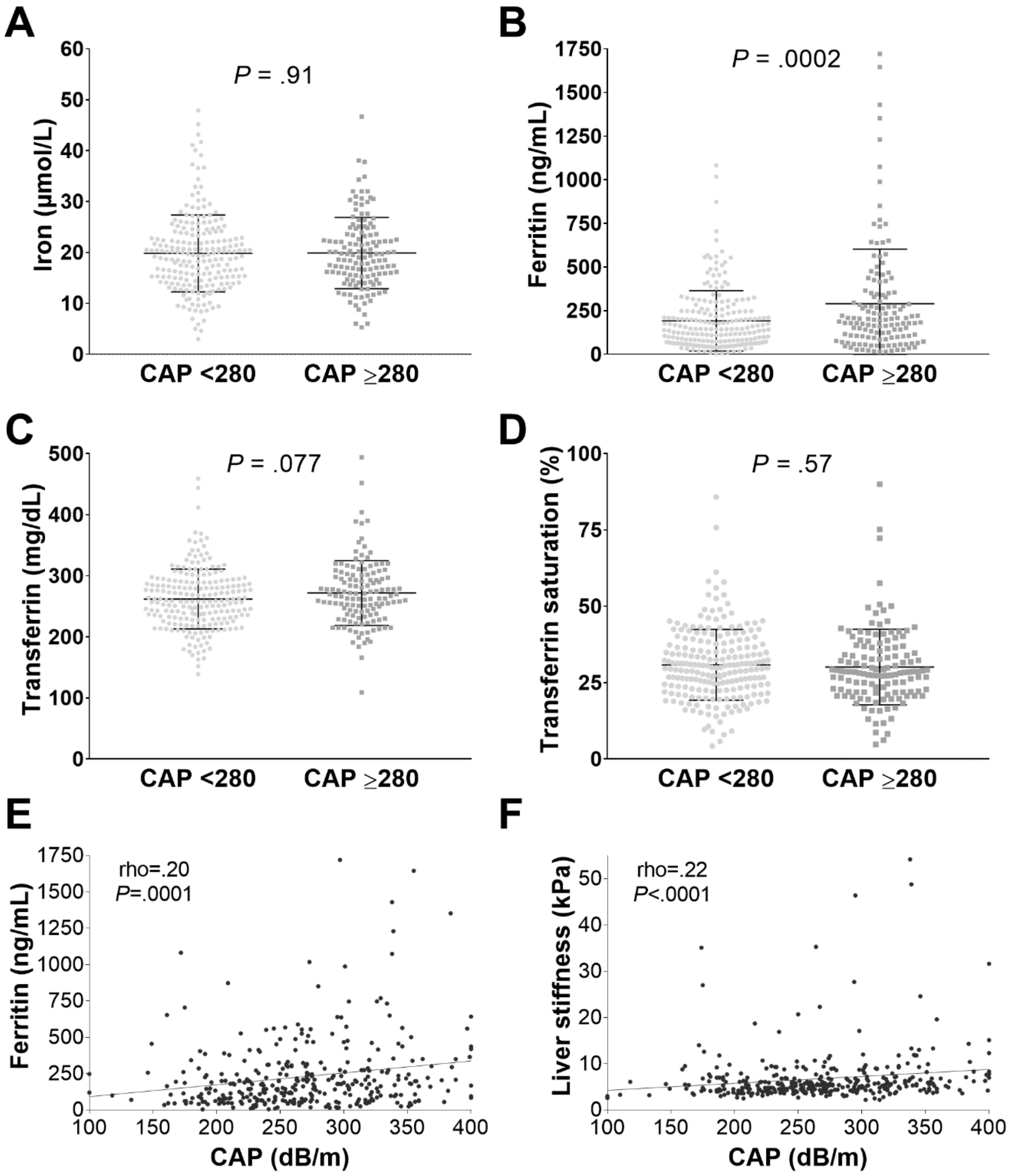

Figure S2. Iron parameters in Pi*ZZ carriers with and without non-invasive signs of severe liver steatosis. Scatter plots of iron (adjusted *P*=0.58) **(A)**, ferritin (adjusted *P*=0.22) **(B)**, transferrin (adjusted *P*=.18) **(C)**, and transferrin saturation (adjusted *P*=0.26) **(D)** in sera of Pi*ZZ subjects with controlled attenuation parameter (CAP) ≥280 dB/m and CAP<280 dB/m, suggestive of the presence/absence of severe liver steatosis. The correlations between CAP and serum ferritin **(E)** as well as liver stiffness **(F)** are shown.

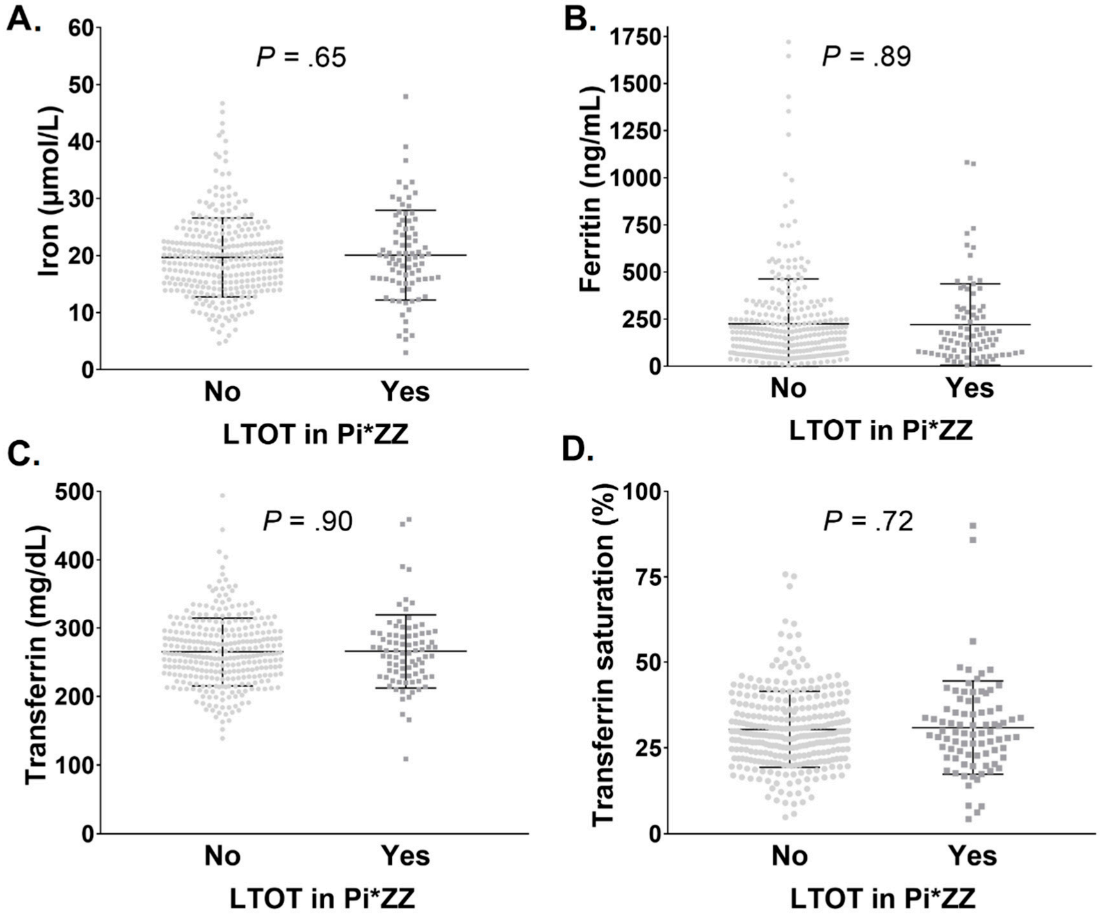

Figure S3. Parameters of iron metabolism in Pi*ZZ individuals with and without long-term oxygen therapy (LTOT). Scatter plots display serum levels of iron **(A)**, ferritin **(B)**, transferrin **(C)**, and serum transferrin saturation **(D)**.

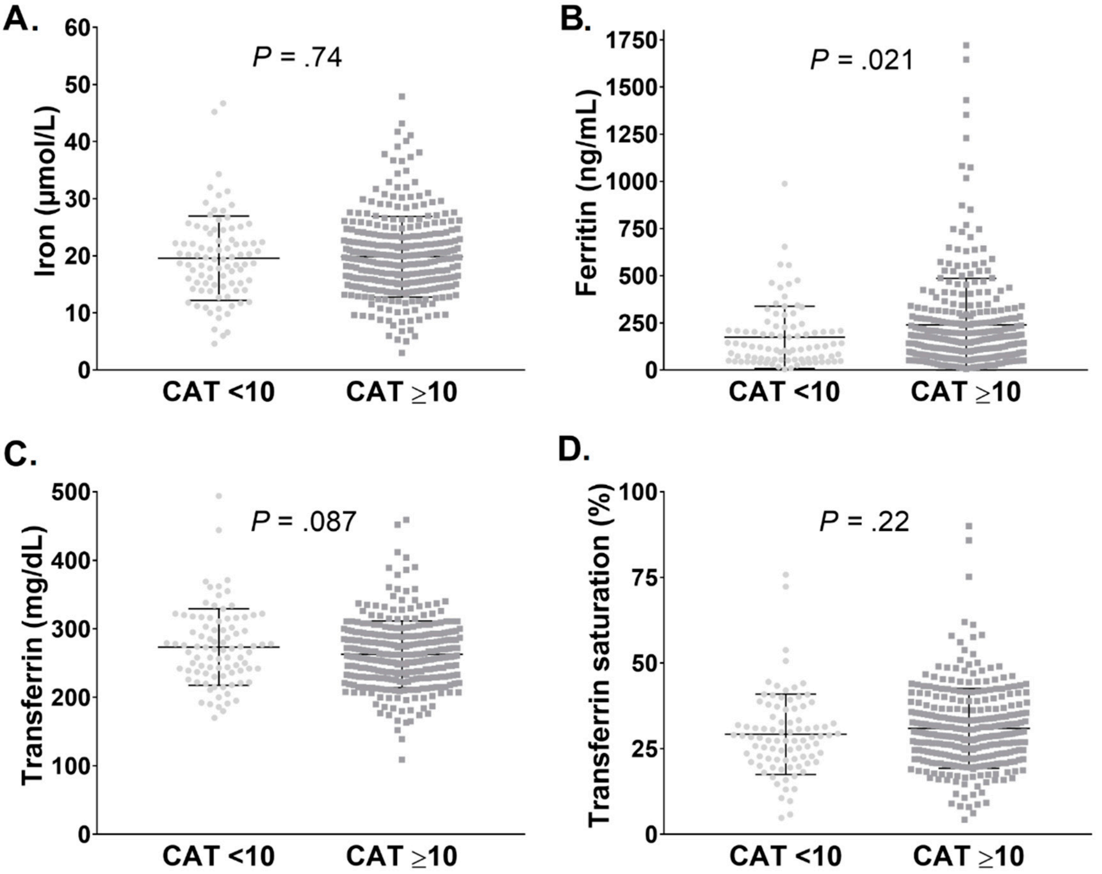

Figure S4. Parameters of iron metabolism in Pi*ZZ subjects with low vs. high COPD assessment test (CAT) scores. Scatter plots display serum levels of iron **(A)**, ferritin **(B)**, transferrin **(C)**, and serum transferrin saturation **(D)**.

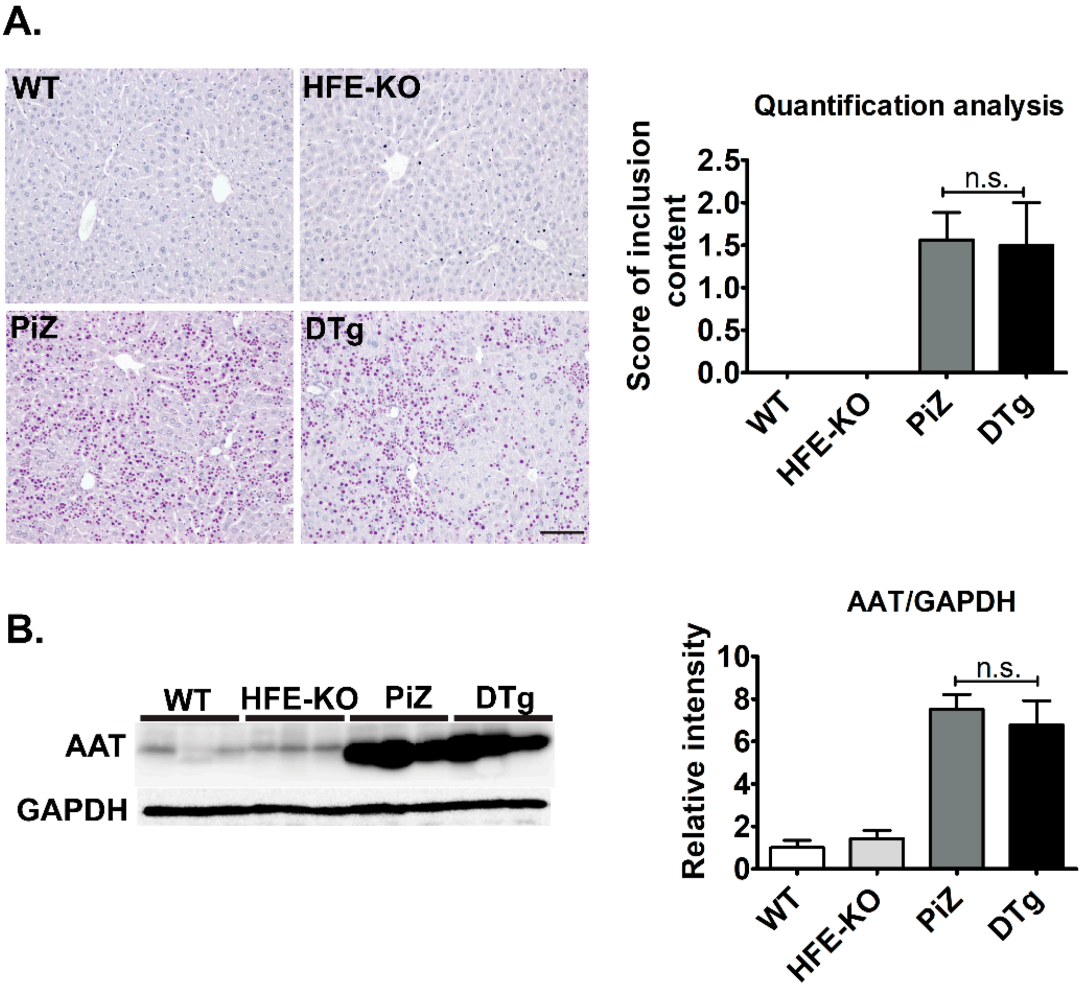

FigureS5. Mild iron overload does not alter the amount of alpha1-antitrypsin (AAT) accumulation in 3 months-old mice overexpressing the Pi*Z variant of AAT. Periodic acid–Schiff–diastase (PASD) staining **(A)** with a morphometric quantification determines the amount of AAT accumulation in non-transgenic (WT) and Homeostatic Iron Regulator knockout (*HFE*-KO) mice, mice overexpressing the PiZ variant of the human AAT (PiZ) as well as double transgenic animals (DTg). Immunoblotting and the corresponding morphometric quantification assessed hepatic AAT levels compared to glyceraldehyde 3-phosphate dehydrogenase (GAPDH) that was used as a loading control (B). Results are shown as mean ± SD (n≥5 per group). n.s., not significant. Scale bar: 100 μm

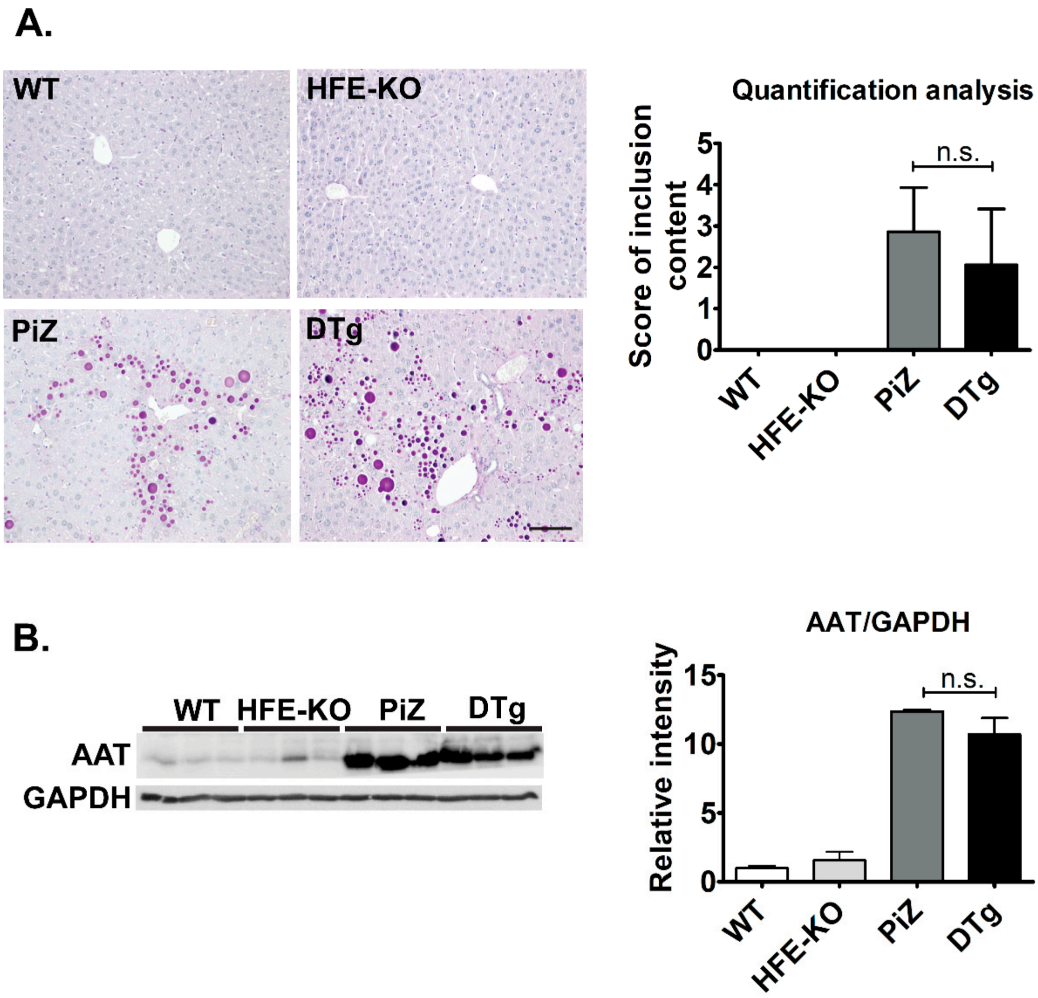

FigureS6. Mild iron overload does not alter the amount of alpha1-antitrypsin (AAT) accumulation in 18 months-old mice overexpressing the Pi*Z variant of AAT. Periodic acid–Schiff–diastase (PASD) staining **(A)** with a morphometric quantification determines the amount of AAT accumulation in non-transgenic (WT) and Homeostatic Iron Regulator knockout (*HFE*-KO) mice, mice overexpressing the PiZ variant of the human AAT (PiZ) as well as double transgenic animals (DTg). Immunoblotting and the corresponding morphometric quantification assessed hepatic AAT levels compared to glyceraldehyde 3-phosphate dehydrogenase (GAPDH), that was used as a loading control (B). Results are shown as mean ± SD (n≥5 per group). n.s., not significant. Scale bar: 100 μm
